# The prism model: advancing a theory of practice for arts and humanities in medical education

**DOI:** 10.1007/s40037-021-00661-0

**Published:** 2021-04-29

**Authors:** Tracy Moniz, Maryam Golafshani, Carolyn M. Gaspar, Nancy E. Adams, Paul Haidet, Javeed Sukhera, Rebecca L. Volpe, Claire de Boer, Lorelei Lingard

**Affiliations:** 1 Department of Communication Studies, Mount Saint Vincent University, Halifax, Nova Scotia Canada; 2 University of Toronto, Toronto, Ontario Canada; 3 Faculty of Health, Dalhousie University, Halifax, Nova Scotia Canada; 4 Penn State College of Medicine, Hershey, PA USA; 5 Woodward Center for Excellence in Health Sciences Education, Penn State College of Medicine, Hershey, PA USA; 6 Departments of Psychiatry/Paediatrics and Centre for Education Research & Innovation, Schulich School of Medicine & Dentistry, Western University, London, Ontario Canada; 7 Department of Humanities, Penn State College of Medicine and Milton S. Hershey Medical Center, Hershey, PA USA; 8 Doctors Kienle Center for Humanistic Medicine, Center Stage Arts in Health, Penn State College of Medicine and Penn State Health Milton S. Hershey Medical Center, Hershey, PA USA; 9 Department of Medicine, Centre for Education Research & Innovation, Schulich School of Medicine & Dentistry, Western University, London, Ontario Canada

**Keywords:** Arts, Humanities, Medical education, Qualitative analysis

## Abstract

**Introduction:**

The arts and humanities have transformative potential for medical education. Realizing this potential requires an understanding of what arts and humanities teaching is and what it aims to do. A 2016 review of exclusively quantitative studies mapped three discursive positions (art as intrinsic to, additive to or curative for medicine) and three epistemic functions (art for mastering skills, perspective taking, and personal growth and activism). A more inclusive sample might offer new insights into the position and function of arts and humanities teaching in medical education.

**Methods:**

Informed by this 2016 framework, we conducted discursive and conceptual analyses of 769 citations from a database created in a recent scoping review. We also analyzed the 15 stakeholder interviews from this review for recurring themes. These three analyses were iteratively compared and combined to produce a model representing the complex relationship among discursive functions and learning domains.

**Results:**

The literature largely positioned arts and humanities as additive to medicine and focused on the functions of mastering skills and perspective taking. Stakeholders emphasized the intrinsic value of arts and humanities and advocated their utility for social critique and change. We offer a refined theory of practice—the Prism Model of four functions (mastering skills, perspective taking, personal insight and social advocacy)—to support more strategic use of arts and humanities in medical education across all learning domains.

**Discussion:**

The Prism Model encourages greater pedagogical flexibility and critical reflection in arts and humanities teaching, offering a foundation for achieving its transformative potential.

**Supplementary Information:**

The online version of this article (10.1007/s40037-021-00661-0) contains supplementary material, which is available to authorized users.

In 2019, the largest scoping review of the arts and humanities in medical education to date was conducted [1]. Led by our team, this review of 769 articles describes the content of these educational initiatives [1]. The volume of articles, however, precluded deeper analyses of the assumptions, values and beliefs underlying these initiatives—questions that have potential to advance the field. This gap creates an opportunity for further, critical study of this literature.

Such critical study is necessary. Because while the arts and humanities have potentially significant and transformative value in medical education [2–7], questions and debates persist about their achievement of that potential value. Educators tend to use the arts and humanities in instrumental ways, often focusing on their utility to teach skills and foster empathy [8, 9]. Because limited evaluation data exist, the impact and effectiveness of arts and humanities teaching has been questioned [10, 11].

Troubling though it is, the effectiveness debate may be premature. Reflecting on the results of their 2016 scoping review of 62 quantitative outcome studies of arts and humanities teaching, Dennhardt et al. cautioned that the effectiveness question is irrelevant until we first understand what arts and humanities teaching *is* and what it is trying to *do* [2]. Their analyses yielded a novel conceptual framework that mapped three discursive positions (art as intrinsic to, additive to or curative for medicine) and three epistemic functions: “art for mastering skills; art for interaction, perspective taking and relational aims; and art for personal growth and activism” [2]. Depicted on a continuum from content- to process-oriented uses, the epistemic functions offered a way to account for critical differences in the assumptions, values and beliefs about how learning from, with and through the arts and humanities occurs. While they offered their framework for scholars to work with in future empirical studies and reviews, Dennhardt et al. recognized that it was constrained by their sampling. The quantitative outcome studies they included “rarely stated their epistemological assumptions explicitly”, leaving the authors “little information to work with” [2] and raising the possibility that a different sample of studies might provide new insights into the discursive positions and epistemic functions of arts and humanities teaching.

Our 2019 scoping review of the arts and humanities in medical education [1] offered a rich data set to explore the assumptions, values and beliefs underlying such educational initiatives using Dennhardt et al.’s framework [2]. The descriptive results portrayed a rich and diverse literature dominated by the literary arts, focused disproportionately on undergraduate medical education, consisting predominantly of program description and conceptual works, authored mostly by medical or health faculty members and lacking in program evaluation, learner assessment and substantive engagement with theory [1]. Overall, while the body of knowledge regarding arts and humanities in medical education was found to be considerable, the descriptive results depicted a fragmented field made up of parallel conversations (i.e., about specific arts and humanities subfields like narrative medicine or specific forms like music), rather than a unified body of developing knowledge regarding what arts and humanities *is* and *does* in medical education [1].

Without such a body of knowledge to draw on, medical educators risk using arts and humanities in limited ways and failing to realize their transformative potential. We anticipated that conceptual and discursive analyses of this large database of published records, informed by Dennhardt et al.’s framework [2], might help us towards a more integrated understanding of what arts and humanities teaching is—and what it can be in the future.

## Methods

We conducted analyses on literature included in a scoping review to address the question: How and why are the arts and humanities being used to educate physician and interprofessional learners across the developmental spectrum?

To contextualize the data set (*n* = 769 articles) analyzed in this paper, we briefly summarize the original scoping review’s inclusion and exclusion criteria and approach to data collection. For complete details, see Moniz et al. [1].

### *Summary of the scoping review methodology*

The scoping review explored a range of arts and humanities used in medical education—from literary and visual arts to history and theology. The data set included English-only results published since 1991, foundational historical works, qualitative and quantitative research, descriptive and conceptual pieces, and research about elective and required programming from premedical to continuing medical education including experiences with interprofessional learners. It excluded grey literature. The data set also excluded empirical and descriptive works about programs outside the United States or Canada but included conceptual papers from other countries that were foundational works in the field and/or addressed the field broadly and with relevance beyond borders [12]. Searches of seven databases were conducted in 2019, locating 21,985 citations. These citations were independently screened, resulting in 769 articles that met inclusion criteria.

### Analysis of epistemic position

Using this data set of 769 articles, we conducted a critical discourse analysis [13] of how the arts and humanities are positioned in relation to medicine, applying the framework developed by Dennhardt et al. [2]. A critical discourse analysis seeks to understand how language constructs the social world [2, 13]. The Dennhardt et al. [2] framework includes three epistemic positions: intrinsic (i.e., a natural, essential part of medicine), additive (i.e., a complement to medicine), or curative (i.e., a remedy that can address shortcomings of medicine and medical education) [2]. We analyzed each article to establish whether the language used in it positioned the arts and humanities as intrinsic to, additive to, or curative for medicine (or ‘other’), guided by the questions: What relationship between the arts and humanities is constructed in this article? What are the arts and humanities in relation to medicine [2]?

### Analysis of epistemic function

We conducted a conceptual analysis [2, 14–17] of epistemic function—that is, what the arts and humanities are *conceived as and therefore used for*—using Dennhardt et al.’s [2] framework. A conceptual analysis seeks to provide clarity around the meaning of a concept by breaking it down into simpler elements and defining its attributes [2, 16]. The Dennhardt et al. [2] framework depicts a continuum from content- to process-oriented foci mapped across three main functions: arts and humanities as expertise (used for mastering skills), arts and humanities as dialogue (used for interaction, perspective taking, and relational aims) and arts and humanities as expression and transformation (used for personal growth and activism) [2]. We analyzed each article to identify how it conceptualized the focus and function of arts and humanities-based teaching and learning, guided by the following questions: “What assumptions framed the teaching intervention? What is arts and humanities-based teaching assumed to be and do? What type of knowledge is sought and how is it assumed to be generated through this type of teaching?” [2]. Once the records were grouped according to Dennhardt et al.’s [2] three functions, we conducted additional qualitative analyses, explained below.

Using content analysis techniques [18], we analyzed the subset of literature focused on using arts and humanities for ‘mastering skills,’ aiming to develop a typology of skills in response to stakeholder interest. We created a preliminary list of ‘types of skills’ informed by the literature [2, 3] (e.g., observational, case presentation/reporting, visual diagnostic, communication, critical thinking, ethical reasoning, metacognitive) and iteratively developed and piloted a data charting form to analyze the literature, guided by the question: What type(s) of skill(s) or knowledge are the arts and/or humanities being used to teach in this record?

We conducted a discursive analysis [13] of a sample of 10% of total records coded as using arts and humanities for ‘interaction, perspective taking, and relational aims.’ We sought to determine whether this subset of literature aligned with the description articulated in the Dennhardt et al. framework [2] or whether there was opportunity to refine it and advance theory in this domain. If the sample suggested potential to further unpack this category, an analysis of the full data set for this function would follow.

Additionally, we conducted a qualitative analysis of literature that used arts and humanities for ‘personal growth and activism,’ both records coded with this single epistemic function and those with multiple epistemic functions that included ‘personal growth and activism.’ In the first round of conceptual analysis, we observed that various examples were being coded into this single epistemic function; we thus aimed to determine whether ‘personal growth’ and ‘activism’ might be teased apart as discernible epistemic functions.

### Stakeholder consultation

We conducted stakeholder consultations concurrently with and informed by the later rounds of analysis in the original scoping review [1]. The research team developed a list of prospective stakeholders based on their reputation in the field as well as gaps identified in the review synthesis [1], namely the absence of artist and medical student perspectives. Our purposive sample [19] thus comprised authors of influential texts, educators overseeing arts- and humanities-based curricula, leaders and administrators supporting curricula, artists, docents and medical students. We developed a semi-structured interview guide (see the Appendix in the Electronic Supplementary Material [ESM]) that included questions about the assumptions that inform the use of arts and humanities in medical education as well as their integration and positioning in medical education. One researcher (PH) conducted the interviews. Participants viewed a summary of results from the scoping review [1] and the discursive and conceptual analyses and then responded to the findings, stating what resonated with their knowledge and experiences and what did not. We met regularly to discuss the interview transcripts, applying techniques of qualitative content analysis [18] to identify patterns and gaps.

This research received clearance from the University Research Ethics Board at Mount Saint Vincent University (#2019-015) and exemption from the Institutional Review Board at Penn State College of Medicine (#STUDY00013567).

## Results

This paper presents a refined theory of practice for the field, which derives from the discursive and conceptual analyses and draws on the stakeholder interviews. Given the large data set (*n* = 769), we cite selected rather than all articles [1] in each category to illustrate findings.

### Discursive analysis of epistemic position

Nearly two-thirds of the literature positioned the arts and humanities as ‘additive’ to medicine [20]. Less frequently, the arts and humanities were positioned as ‘curative’ to medicine [21] and few records positioned the arts and humanities as ‘intrinsic’ to medicine [22]. A few records had no epistemic position. Occasionally a record invoked more than one epistemic position, with ‘additive and curative’ being the most common pairing.

### Conceptual analysis of epistemic function

Nearly half the records used arts and humanities to either ‘master skills’ [23] or, equally, to promote ‘interaction, perspective taking and relational aims’ [24]. Less frequently, arts and humanities were used to promote ‘personal growth and activism’ [25]. A few records invoked no epistemic function, notably descriptive records about arts and humanities programming [26]. Rarely did records have ‘other’ epistemic functions, such as bringing joy [27]. More than a third of the records involved more than one epistemic function, with ‘mastering skills’ and ‘interaction, perspective taking and relational aims’ being the most prevalent pairing [28]. Of remaining records, most used arts and humanities for either ‘interaction, perspective taking and relational aims’ and ‘personal growth/activism’ [29], as well as for all three functions combined (*n* = 84, 29%) [30]. Few records used arts and humanities for both ‘mastering skills’ and ‘personal growth and activism’ [31].

#### Arts and humanities for mastering skills: Attempting a skills typology

Our attempt to create a typology of skills by analyzing all records that used arts and humanities for mastering skills was ultimately unsuccessful for three main reasons. First, a lack of coherence in language use and definitions of concepts made reliable categorization difficult. For instance, terms like ‘skills’ and ‘learning outcomes’ were often used inconsistently or interchangeably across the records, making it difficult to reliably assign records to a category. Second, the focus on skills necessarily excluded several important domains that the arts and humanities were used to teach that cannot be categorized as ‘skills,’ such as equity, diversity and inclusion. Third, some of the most common ‘skills’ in records coded within the first epistemic function, such as communication, were also treated as more-than-just-skill in records coded with other functions. For example, communication was also the focus of some records using arts and humanities for the second function of ‘interaction, perspective taking and relational aims’ [32, 33] and the third function of ‘personal growth and activism’ [34, 35]. Therefore, we concluded that a typology of skills would offer, at best, an unstable, incomplete and oversimplified categorization of this rich literature.

#### Arts and humanities for dialogue: Replicating Dennhardt et al.’s findings

Our discursive analysis of the sample of records that used arts and humanities for interaction, perspective taking and relational aims aligned with the description of this function articulated in the Dennhardt et al. framework [2], leading us to conclude that there was nothing further to unpack in this domain.

#### Arts and humanities for personal growth and activism: Refining Dennhardt et al.’s framework

Our analysis of records categorized into Dennhardt et al.’s [2] third epistemic function found two categories of records: those focused on using arts and humanities for personal insight into emotions and professional identity [36] and those focused on instilling awareness of inequities, civic-mindedness and advocacy for transformational change in healthcare and society broadly [5]. We therefore split Dennhardt et al.’s [2] third epistemic function, creating a revised model of four epistemic functions of arts and humanities in medical education: arts and humanities for mastering skills, arts and humanities for perspective taking, arts and humanities for personal insight, and arts and humanities for social advocacy. Table 1 outlines the distinctions between the third and fourth functions in this revised model.

**Table 1:** Epistemic function of arts and humanities teaching for ‘personal insight’ and for ‘social advocacy’ in medical education (adapted and revised from Dennhardt et al. [2])

Epistemic function	Arts and humanities for personal insight	Arts and humanities for social advocacy
*Function of arts and humanities that is invoked*	Arts and humanities are expression and can be used for emotional growth, professional identity formation and wellbeing	Arts and humanities are advocacy and can be used for socio-cultural critique and change
*Assumption of what arts and humanities do and how arts and humanities create knowledge*	Engaging with and/or creating art and engaging with the humanities allows students to gain insight into, express and deal with their emotions Arts and humanities can support and protect learners in finding meaning in medicine Arts and humanities can counteract and transform a perceived ‘lifelessness’ of a dominant technical science	Engaging with and/or creating art and engaging with the humanities allows students to question ‘ways of seeing’ or ‘being’ that are dominant, even oppressive, in medicine, including in medical culture, institutions and systems and including their own ways of ‘seeing’ or ‘being’ that perpetuate such systems Arts and humanities make visible and can change social injustices in medicine
*Focus of where arts and humanities do what they do*	Focus on making meaning of the self in the context of a career in medicine	Focus on future physicians and their role in contexts of cultures, systems and institutions
*For whom*	Arts and humanities to express and make sense of human experience and emotions; arts and humanities for personal growth and to humanize doctors and medicine	Arts and humanities to improve broader systemic challenges
*Type of knowledge emphasized*	Knowledge about the self and about ‘how to become’ (and what it means to be) a physician	Knowledge about how medical culture, systems and institutions benefit some and disadvantage others, and about how one may be complicit with or critical of culture, systems and institutions
*Main attention*	Attention to one’s own emotions and experiences through medical training and practice	Attention to what needs to be changed in medicine (e.g., health disparities), made visible or brought to light
*Example*	A mask-making workshop to promote self-reflection, professional identity formation and self-care	An art exhibit to critically engage with issues of bias and stigma in patient care
*Typical language terms*	Wellbeing, wellness, burnout, self reflection, meaning, becoming, growth, emotions, expression, resilience	Critical reflection, social (in)justice, society, diversity, equity, inclusion, assumption(s), bias(es)

We selected ‘advocacy’ rather than ‘activism’ as the label for the fourth category, as it better reflected these records’ emphasis on “social conscience and attention to systems of inequality, power, and privilege and working to eliminate social inequities and injustice in the interest of the common good” [37]. This literature included articles on addressing systemic and physician bias through empowering and educating physicians about the inherent biases within the system that privilege normative ways of thinking, being and treating patients [38]. Separating social advocacy from personal insights further resonates with a growing literature focused on the orientation of the medical humanities for social justice aims [7].

### Stakeholder interviews

We interviewed 15 stakeholders: 3 educators of arts- and humanities-based curricula, 4 administrators supporting arts- and humanities-based curricula, 2 artists, 1 museum educator, 2 medical learners, and 3 scholars. Some participants represented more than one category. Three were also practicing clinicians.

Interviewees reported that the positioning in the literature of the arts and humanities as additive to medicine resonated with their experience. Many lamented that the field does not appreciate how ‘intrinsic’ the arts and humanities are to medicine. One medical learner found it

*frustrating because basically the way that *[arts and humanities]* programs are constructed is that they’re optional most of the time and they’re not built into the curriculum. They’re not considered an important intrinsic part of the curriculum a lot of the time …* – P7

Some perceived that the additive emphasis reflects an underlying hidden curriculum which a medical student stakeholder labelled “performative humanism,” calling attention to institutional treatment of the arts and humanities as a type of “window dressing on the program” (P2). Others perceived that the additive position promoted a related assumption that teaching arts and humanities curricula is, as one docent put it, “easy” and that “anybody can do this kind of work” (P1). A faculty member affirmed:

*If we really value the voice of the humanities and social sciences in terms of what they contribute to healthcare, then shouldn’t we be pulling it more into the centre or integrating it across so it doesn’t seem as if it’s an add on?* – P13

While many interviewees expressed concern at the additive positioning and its effects, however, they also acknowledged that they participate in this positioning by failing to speak about arts and humanities as ‘intrinsic’ in their own educational contexts. An administrator of a medical humanities program reflected on their own tendency to write about and approach arts and humanities programming as “enrichment opportunities,” calling for the need to make arts and humanities more intrinsic and calling out “institutional buy-in, budget” as a challenge to doing so: “I think that in general a lot of people in high positions don’t understand why [arts and humanities] are important and necessary” (P3). Rather than advocate at an institutional level for why arts and humanities are integral to medical education, participants described how they turn to informal methods of talking about arts and humanities within medicine, such as Twitter, which exacerbate its additive positioning.

With respect to the functions of the arts and humanities in medical education, stakeholders acknowledged the literature’s emphasis on the first two functions – mastering skills and perspective taking – as a faithful, albeit worrisome, representation of their experience. Some interviewees spoke disparagingly about the tendency for educators to view the arts and humanities as tools to help learners develop expertise or build skills. One reflected:

*I wish more people felt that *[arts and humanities]* contributed to this other part of themselves—to better articulate themselves, to better mirror themselves and to have an expansion of inner space …. I think a default mode is often expertise …* – P8

Many stakeholders advocated for a less instrumental view of arts and humanities in medical education. In particular, interviewees perceived the arts and humanities as having real utility for social critique and change, including around health disparities. As one administrator said:

*For me, *[critical medical humanities]* means thinking about humanities scholarship and relationships with the marginalized communities, … all of these things that are greater problems in our healthcare system that we don’t really talk about, and how that has shaped our system and the way that we think in a way that so many of us don’t even question where we are …* – P3

Interviewees also called for medical educators to broaden their view of the subject or concept they are trying to teach, such as social justice, empathy or communication. An artist said:

*If we don’t know about racial inequity and the continual perpetration of structural violence on people’s colour, you can’t fully receive the person in front of you for the person that they are. So, for me, social justice and medicine are interwoven, and I would expect that medical education that seeks to be inclusive and truly humane … address*[es]* this intermixing complex terrain …* – P6

The need for an overarching theory of practice was also articulated by multiple stakeholders as necessary to develop the role of arts and humanities in medical education. For instance, a docent stated: “We do need to build a theory of practice to give it some real root[s] …. [H]ow do we theorize this work that we do?” (P1) Repeatedly, interviewees emphasized the need to be thoughtful and critical about the function of the arts and humanities in order to realize its potential for the field of medicine.

## Discussion

Our application of Dennhardt et al.’s [2] framework to 769 published works has resulted in a refined model of the position and function of arts and humanities teaching in medical education. We use this model as the basis for a theory of practice that would assist educators to achieve the full transformative potential of their arts and humanities teaching.

Our refinement of Dennhardt et al.’s [2] model from three functions to four creates a distinction between arts and humanities for personal insight and arts and humanities for social advocacy. This development was likely possible because our inclusion criteria were more expansive than Dennhardt et al.’s [2]: we included any research type and methodology, whereas they included only empirical studies using quantitative methods to evaluate outcomes. Stakeholders resonated with the refined four-function model, in part because explicit invocation of social advocacy resonates with medical education’s current aim to develop learners’ advocacy.

More important than our shift from three to four functions, however, is our reconceptualization of their relationship. Dennhardt et al. conceptualized the epistemic functions as existing in linear relationship, along a “continuum that extends from content- to process-oriented uses” [2]. While the continuum metaphor acknowledges overlap, it nevertheless implies that any particular arts and humanities study occupies a point on that continuum, an implication strengthened by the reflection that this continuum of epistemic function “runs parallel with the continuum of research methodologies from post-positivist [to] social constructivist and critical [theoretical]” [2]. Informed by a larger, more diverse sample, we reconceptualize the functions not as a linear continuum but as a dispersive prism. This is not simply wordplay. Our intention is for the model to help educators to tackle any topic in multiple ways depending on which function is foregrounded, rather than to align with one function over another: the metaphor of a prism, rather than a continuum, captures that difference for us. We intend this model to serve as a meaningful and practical pedagogical guide for educators, one that enriches how they ‘see’ the possibilities for the arts and humanities as they imagine, plan, execute and evaluate their educational initiatives.

An explanatory model of the four functions of arts and humanities—the Prism Model—is at the center of our proposed theory of practice. This model seeks to explain how a learning domain may be differently taught according to the epistemic function—that is, what the educator’s purpose is. We use the term ‘learning domain’ to refer to the subject matter, whether this is communication or social justice or something else. Regardless of learning domain, all four functions are potentially relevant, and educators can cultivate pedagogical flexibility by thinking critically about how each of the four epistemic functions would shape their approach to a particular learning domain. Fig. 1, found in the Electronic Supplementary Material (ESM), demonstrates how such pedagogical flexibility would work using the exemplar of teaching social justice.

The Prism Model provides a vocabulary and a process for educators to reflect on how each function emphasizes some aspects of a learning domain and elides others. The four functions are most powerful in combination, as a method to refract the spectrum of pedagogical possibilities. By applying the Prism Model to explore the affordances and limitations of each approach to teaching a learning domain, educators can strategically select the approach, or combination of approaches, that best fits their organizational culture, their own values and their curricular resources. The functions are not hierarchical: one is not ‘better’ than the others, nor is one necessarily the ‘right’ way to teach a learning domain. Each function offers a ‘way of seeing,’ selecting some things for our attention and deflecting our attention from others [39]. While the epistemic function of social advocacy might seem an obvious fit for the learning domain of social justice (just as the function of mastering skills might seem an obvious fit for the learning domain of communication), it may not be appropriate depending on several factors. These include the position of the learner on the physician development trajectory; the facilitator’s skills and background, collaborative relationships and available resources; and the extent to which the epistemic function requires pedagogy that reflects or resists the current organizational climate. The last factor is rarely explicitly discussed but is not to be underestimated. Teachers should reflect on whether their arts and humanities approach puts them into a position of resistance vis-à-vis their dominant organizational climate [40], so that they can strategically position their efforts, support their students and navigate their own potential experience of dissonance [41].

The main limitation in this analysis derives from the tacit nature of the epistemological positioning in this literature. As also noted by Dennhardt et al. [2], authors are seldom explicit in articulating the assumptions underlying their use of arts and humanities in medical education, which meant we relied on qualitative methods to infer meaning from their uses of language. This creates the possibility for misattribution. Furthermore, while the sample of stakeholders interviewed was purposeful, it was also small and comprised of ‘insiders’ to the field. For a discussion of the limitations related to the creation of the database in the original scoping review, see Moniz et al. [1].

## Conclusion

The field of arts and humanities in medical education has tremendous diversity and potential. This potential remains unrealized, however, in the absence of a theory of practice that can inform—and be informed by—the efforts of educators in all the subfields working with any learning domain. Offering critical refinements to previous work, we offer the Prism Model to assist medical educators to orient their arts and humanities initiatives more purposefully, according to which epistemic functions map onto their institutional cultures, their professional values, their resources and their educational aims. Any course could benefit from this level of critical scrutiny. We hope that by encouraging greater pedagogical flexibility, critical reflection and strategic planning, this theory of practice can offer a basis for advancing a shared vision that reflects the transformative potential of arts and humanities in medical education.

## Supplementary Information

Appendix

Figure 1 Teaching social justice using the Prism Model
