# Supplementary material for: The prism model: advancing a theory of practice for arts and humanities in medical education
Source: Perspect Med Educ. 2021 Apr 29;10(4):207–14. doi: 10.1007/s40037-021-00661-0 (PMC8368821; doi:10.1007/s40037-021-00661-0)
Supplement: Supplementary file 1 — Appendix [file 40037_2021_661_MOESM1_ESM.pdf]

**The Prism Model:**
**Advancing a theory of practice for arts and humanities in medical education**

Tracy Moniz, Maryam Golafshani, Carolyn M. Gaspar, Nancy E. Adams, Paul Haidet, Javeed Sukhera, Rebecca L. Volpe, Claire de Boer, Lorelei Lingard

## INTERVIEW GUIDE

### Part 1: Response to Results

- What general reactions do you have to these results (i.e., from the scoping review,[1] from the discursive and conceptual analyses)?
  - Probe specific reactions, as necessary.
- How do these results intersect with your own experience in teaching/learning the humanities in health sciences settings?
  - Probe for specific stories about how results played out in the interviewee's experience.
- What, if anything, does NOT ring true for you in these results?
  - Probe as necessary
- These are the gaps we identified in the literature (name gaps).
  - Do these gaps ring true to you?
  - Are there any additional gaps in this literature, from your perspective, that you see as important?
  - What implications do such gaps have for using the arts and humanities in health sciences education?

### Part 2: Additional Interview Topics

**GAPS**: We will ask questions that explore the gaps in knowledge related to teaching the arts and humanities in medical education. For instance:

- What gaps in our knowledge exist with respect to teaching the arts and humanities in medical education?
- Why do you think that certain gaps exist in our knowledge/the literature?
- What does this community need to focus on that it currently, from your perspective, does not focus on?

**CHALLENGES:** We will ask questions related to threats and/or challenges in teaching the arts and humanities in medicine. For instance:

- What do you see as the greatest threats to teaching the arts and humanities in medical education?
- What do you see as the greatest disappointments or failures in teaching the arts and humanities in medical education?

[1] Moniz T, Golafshani M, Gaspar C, Adams NE, Haidet P, Sukhera J, Volpe RL, de Boer C, Lingard L. 2021. How are the arts and humanities used in medical education?: Synthesis results of a scoping review of published literature. *Academic Medicine,* forthcoming.

**CONCEPTUAL:** We will ask questions that explore stakeholder perspectives on whether the 'arts' and the 'humanities' occupy the same conceptual "space." For instance:

- Do you think the 'arts' and 'humanities' occupy the same conceptual "space" and serve the same purposes and objectives in medical education, or are they distinct? Explain.
- What connections do you see between the 'arts' and 'humanities'? What differences do you perceive?

**RESOURCES:** We will ask questions about resourcing to teach arts and humanities in medical education. For instance:

- Is there dedicated funding for arts and humanities programming in medical education in your setting? What is the funding model?
- What kinds of initiatives/programs/efforts are resourced well in arts and humanities curricula?
- What kinds of initiatives/programs/efforts need better resources? What do you struggle to get resourced?
- What kinds of administrative support exists/needs to exist?

**LEADERSHIP:** We will ask questions related to administrative leadership in arts and humanities in medical education. For instance:

- Is there dedicated leadership for arts and humanities programming in medical education in your setting?
  - Is arts and humanities programming part of someone's job description or do such efforts happen on the fringe?
  - What is the impact of this organizational structure, from your perspective? (e.g., on faculty? curriculum? learners?)

**EDUCATIONAL CONTENT**: We will ask questions about the content of arts and humanities programming/curriculum in medical education. For instance:

- What kinds of subjects should be taught using the arts and humanities in medical education?
- What types of learning objectives/assumptions/intentions inform the use of arts and humanities in medical education?
- What types of outcomes should be measured/assessed?
- What do you see as the multiple goals of the arts/humanities, and what kinds of tensions exist between such goals?
  - What implications do such tensions have for the impact of arts and humanities in medical education?

**INTEGRATION:** We will ask questions about integrating the arts and humanities in medical education. For instance:

- How are the arts and humanities integrated (e.g., as elective or required programming/curriculum, etc.) in your setting? How did it come to be that way in your institution?
- What kinds of conversations occur (e.g., in curriculum committees, etc.) about the arts and humanities in your institution?
- Speak to the idea of arts and humanities as recreational learning (i.e., with no curricular credit)
    - Does it happen this way in your institution? Why or why not?

**POSITIONING:** We will ask questions about the position of the arts and humanities in medical education. For instance:

- How central is/should the arts and humanities be in medical education?

**APPRECIATIVE INQUIRY:** We will ask questions that probe stakeholders' perceptions and experiences about 'best practices' in teaching the arts and humanities in medical education. For instance:

- What currently works well with respect to teaching the arts and humanities in medical education? Why?

**FACULTY:** We will ask questions about faculty who teach the arts and humanities in medical education. For instance:

- Who teaches arts and humanities in your setting? (e.g., artist-educators, faculty educators? others?)
- Who are the ideal faculty to teach arts and humanities in medical education? Why?
- What kinds of resources should be devoted to such faculty?
- Where should such faculty be housed (i.e., administratively)? What kinds of departments exist/should exist to support arts and humanities in medical education?
