# Supplementary material for: The prism model: advancing a theory of practice for arts and humanities in medical education
Source: Perspect Med Educ. 2021 Apr 29;10(4):207–14. doi: 10.1007/s40037-021-00661-0 (PMC8368821; doi:10.1007/s40037-021-00661-0)
Supplement: Supplementary file 2 — Figure 1 Teaching social justice using the Prism Model [file 40037_2021_661_MOESM2_ESM.pdf]

Figure 1 | Teaching social justice using the Prism Model

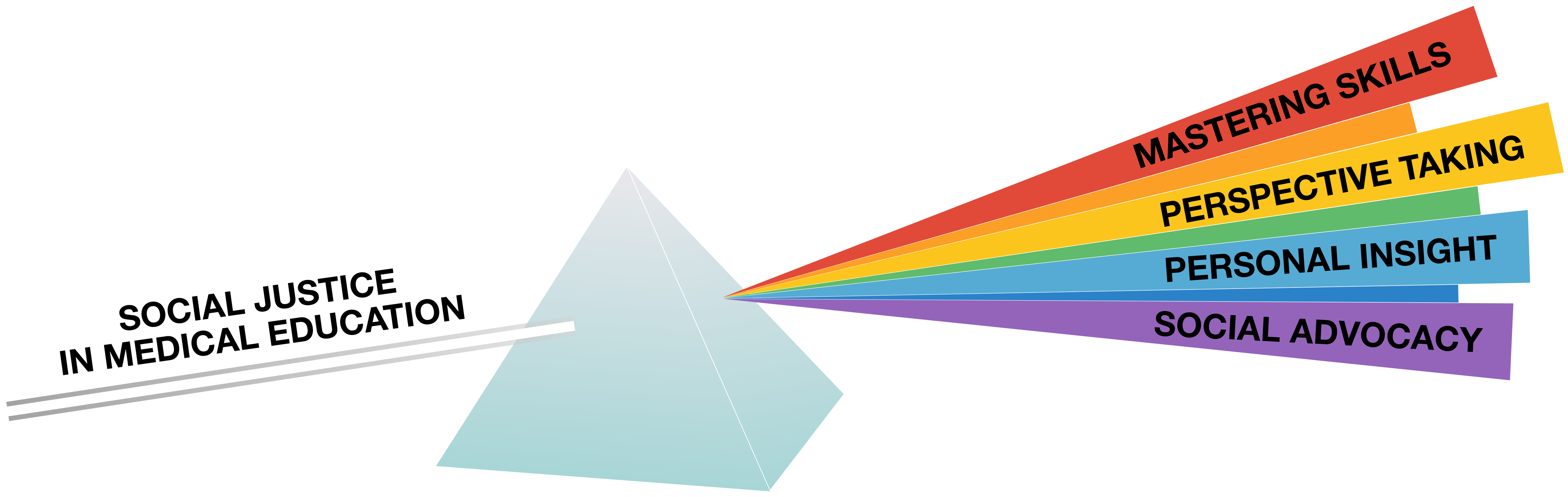

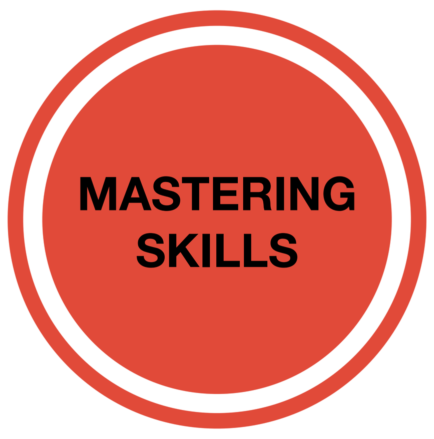

MASTERING SKILLS

In teaching social justice using a **mastering skills** approach, educators would use art and the humanities to help physician learners develop knowledge about social justice or skills that help promote it. Knowledge about culture, justice and power may be taught alongside their impacts on health, health beliefs and healthcare access.[1] This approach is amenable to medical education's current competency-based focus, which may foster students' recognition and appreciation of the relevance of social justice topics to medicine. However, this approach implies that social justice can be achieved by acquiring certain knowledge or skills. To minimize this implication, educators could elaborate their approach to include one or more of the other epistemic functions

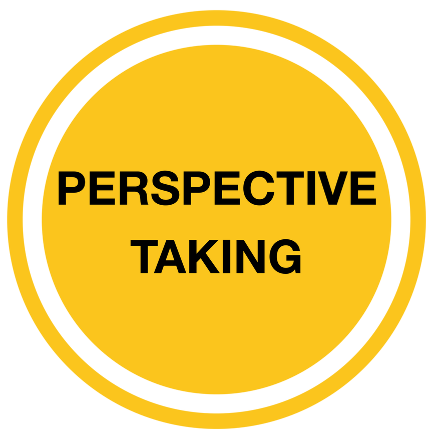

PERSPECTIVE TAKING

In teaching social justice using a **perspective taking** approach, educators would use art and the humanities to build relationships and promote understanding of others' viewpoints, notably those of marginalized patients. Arts-based learning experiences may build awareness of (i.e., through photography that tells a patient's story) and prompt reflection on (i.e., through reflective writing) disparities and injustices in patient care.[2] This approach considers diverse perspectives and promotes empathy. However, this approach assumes that one *can* take the perspective of another person. To encourage learners to consider their own roles in perpetuating oppression or advocating for change, educators could consider approaching aspects of the curriculum through the other epistemic functions.

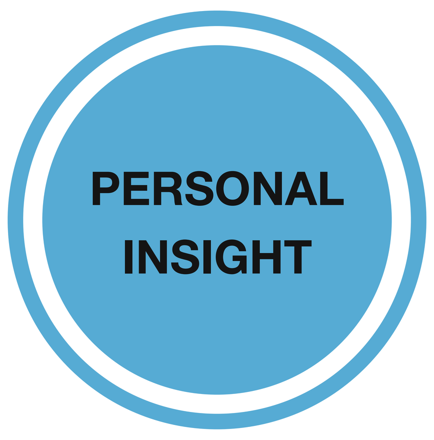

PERSONAL INSIGHT

In teaching social justice using a **personal insight** approach, educators would use art and the humanities to foster critical reflection about social justice in ways that support professional identity formation, resilience, and personal transformation. Art and/or humanities activities (i.e., watching and discussing operas) may be used to engage with aspects of social justice, such as power and the physician-patient relationship as well as stigma and stereotyping of patients with certain diseases, and to facilitate insight into learners' attitudes, behaviours, and experiences (i.e., through reflective writing).[3] This approach focuses on internal barriers to social justice. However, to consider environmental or cultural barriers, educators could also integrate other epistemic functions as part of their approach.

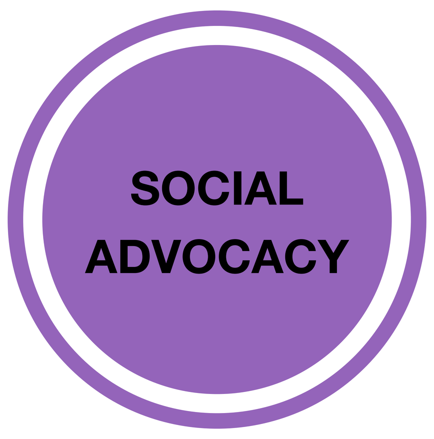

SOCIAL ADVOCACY

In teaching social justice using a **social advocacy** approach, educators would use art and the humanities to foster socio-cultural critique and societal transformation through advocacy. Such learning experiences enable critical analysis of inequities (i.e., through observing and critiquing art and film) as well as exploration of advocacy and intervention in medicine (i.e., through creating and curating an art exhibit focused on patient and provider experiences of bias and stigma).[4] This approach may prompt critical thinking about medical culture and society in the context of medicine and propel action to illuminate and address disparities. However, to ensure learners reflect on their individual role in perpetuating injustice, educators should incorporate other epistemic functions in their teaching.

References:

1. Kuper A, Veinot P, Leavitt J, Levitt S, et al. Epistemology, culture, justice and power: Nonbioscientific knowledge for medical training. *Med Educ.* 2017;51(2): 158-173. doi: 10.1111/medu.13115
2. Ross PT, Lypson ML. Using artistic-narrative to stimulate reflection on physician bias. *Teach Learn Med.* 2014;26(4): 344-349. doi:10.1080/10401334.2014.945032
3. Levin SR, Cai F, Noronha N, et al. Diseases, doctors, and divas: Cultivating reflective capacity in preclinical medical students through a critical examination of opera. *Journal for Learning through the Arts.* 2017;13(1). doi: 10.21977/D913128321
4. Jones T. Moving Pictures: Visual culture/visual activism in the health humanities classroom. In: Olivia Banner et al, ed. *Teaching Health Humanities.* New York, NY: Oxford University Press; 2019
